# Point-of-care detection of *Escherichia coli* O157:H7 in water using AuNPs-based aptasensor 

**Published:** 2020-07

**Authors:** Vahid Soheili, Seyed Mohammad Taghdisi, Khalil Abnous, Mohsen Ebrahimi

**Affiliations:** 1 Department of Pharmacology and Toxicology, Faculty of Medicine, AJA University of Medical Sciences, Tehran, Iran; 2 Targeted Drug Delivery Research Center, Pharmaceutical Technology Institute, School of Pharmacy, Mashhad University of Medical Sciences, Mashhad, Iran; 3 Pharmaceutical Research Center, Pharmaceutical Technology Institute, School of Pharmacy, Mashhad University of Medical Sciences, Mashhad, Iran

**Keywords:** Aptamer, Aptasensor, AuNPs, Escherichia coli O157:H7, Water

## Abstract

**Objective(s):**

Access to safe drinking and irrigation water has always been one of the major human concerns worldwide. Thus, rapid, sensitive, and inexpensive approaches for pathogenic bacteria detection, such as *Escherichia coli *O157:H7 (EHEC) that can induce important infectious diseases, are highly on demand.

**Materials and Methods:**

In this study, a sensitive aptamer-based AuNPs bioassay was developed that demonstrated its potential to detect EHEC. In the presence of the target bacterium, the specific adsorbed aptamer, leaves AuNPs surface and interacts with EHEC. The bare nanoparticles aggregate in the presence of NaCl and the color shifts from red to purple and blue depending on the bacterial concentration.

**Results:**

The proposed aptasensor exhibited a good linear response over a wide concentration range of 876 to 107 CFU/ml and was closely correlated with the line equation of “y=0.0094x+0.0006” (R2= 0.9861). It also showed a low detection limit (LOD) of 263 CFU/ml (Signal/Noise=3). No response was recorded in the presence of other tested bacterial strains including *Listeria monocytogenes* and *Salmonella typhi*, indicating the high selectivity of the aptasensor.

**Conclusion:**

This biosensor may be used as a smart device to screen water reservoirs and prevents the outbreak of EHEC-related life-threatening contagious diseases.

## Introduction

Globally, infectious diseases are responsible for almost 40% of the estimated annual 50 million deaths. Detection and identification of microbial pathogens are especially critical in the area of clinical diagnosis, water and environmental analysis, food safety, and biodefense (1). Every year, millions of people become sick and some of them die as a result of unsafe food and water consumption (2). It has been known that more than 200 various diseases are transmitted through unsafe food (3). Based on the World Health Organization (WHO) reports, foodborne diarrheal diseases commonly caused by gastrointestinal infections, have been responsible annually for the death of almost 2.2 million people (4, 5). Diarrhea is caused by pathogenic micro-organisms such as bacteria, viruses, and parasites and most of them can be spread via contaminated water (5). The contamination of drinking water can be due to ineffective water treatment practices, biofilm formation, leaking of circulation pipelines, and sewage contamination. However, the main risk to human health derives from exposure to water contaminated with pathogen-loaded feces, directly or indirectly (4). Therefore, evaluation of water quality and rapid identification of pathogenic micro-organisms are great challenges to protection of public health and prevention of bioterrorism (6).

In recent years, outbreaks of foodborne illnesses related to a pathogenic bacterium, *Escherichia coli* (*E. coli*), have been widespread and turned into a public health problem (7, 8). Indeed, most of our knowledge about pathogenic *E. coli* originates from outbreak studies of a specific serotype,* E. coli *O157:H7 (EHEC) infection which can cause watery life-threatening diarrhea, hemorrhagic colitis, hemolytic-uremia syndrome (HUS), and thrombotic thrombocytopenic purpura (TTP), particularly in young children and the elderly due to the production of verotoxins (4, 7, 9). The infectious dose of EHEC bacterium is significantly lower than other pathogenic strains of *E. coli* (as low as 100 EHEC cells). Infections of humans by EHEC O157:H7 can take place via direct contact with animal carriers or their feces, consumption of undercooked or raw ground beef, as well as contaminated water, milk, vegetables, and fruits (4, 10). Based on the Centers for Disease Control and Prevention (CDC) report, from 2003 to 2008, EHEC infections were responsible for 32 outbreaks in the United States such as large outbreaks resulting from consumption of contaminated spinach and iceberg and romaine lettuce (7).

To detect and identify microbial pathogens both conventional assays (including microbial culture-based tests and colony counting) and novel molecular assays (including immunological technologies like enzyme-linked immunosorbent assay (ELISA) and radioimmunoassay (RIA) method, or nucleic acid technologies like nucleic acid-based polymerase chain reaction (PCR) and microarray technology) are used (1, 7, 11). Generally, the detection method should be robust and sensitive to reveal targeted pathogens rapidly and quantify them accurately through a proper assay suitable for evaluation of the microbiological quality of water (4). However, conventional detection methods suffer from lengthy processes and lack of precision and sensitivity (9, 12). Compared to the culture-based methods, modern approaches have better sensitivity and specificity but face some limitations (4). For instance, the PCR-based procedures cannot reach low detection limits without a complex set up and proper enrichment culturing (9, 12)**. **They also cannot distinguish between viable cells and dead ones which results in false-positive outcomes (13). Moreover, PCR-based methods are laborious, time-consuming, and require specialized reagents, and expensive complex instruments besides expert operators (14, 15). On the other hand, although immunoassays are very sensitive special conditions are required to prevent denaturation of antibodies during storage and handling, apart from the difficult and expensive process of antibody production in animals and their purification (11, 15). Therefore, the critical problems such as sensitivity, specificity, complexity, assay time, cost involved, limit of detection, besides the necessity to develop a simple and economical device with the ability of on-site monitoring has resulted in employing nanotechnology-based approaches due to the unique physicochemical properties of nanomaterials (4, 16).

The emergence of aptamers (single-stranded DNA or RNA oligonucleotides with randomized sequences capable of folding into three-dimensional structures and recognizing their targets with high specificity) and their wide application in sensing technologies have provided possibilities to couple aptamers with nanoparticles for supplying various biosensors to target and detect pathogenic bacteria, specifically (4, 17, 18). Subsequently, biosensors convert the selective interaction of target and aptamer to a measurable signal. Moreover, the use of aptamers can overcome the disadvantages of antibodies such as thermal and chemical stability, batch-to-batch variation, complexity of synthesis and labeling, cross-reactivity, and cost of production (15, 17-19).

Among different analytical techniques, the colorimetric method is very attractive for bacterial pathogen detection due to its simplicity, practicability, and applicability in a wide dynamic range without the need for sophisticated instruments (18, 20). 

In the present study, we developed an aptamer-based biosensor for simple and rapid detection of EHEC in contaminated water. A single-stranded DNA aptamer specific for detection of the pathogen was selected. Then, a simple and reliable colorimetric aptasensor was established based on the color change of gold nanoparticles (AuNPs) depending on the resulting AuNPs size, without any pretreatment steps such as pre-culturing or cell lysis.

## Materials and Methods

***Chemicals and materials***

In addition to EHEC (NTCC 12900), a Gram-negative bacterium,* Salmonella typhi* (ATCC 1609), and a Gram-positive bacterium, *Listeria monocytogenes* (PTCC 1298) were used as controls. All aptamer sequences used in this study were synthesized and purified by Bioneer Company (South-Korea) (Table 1). Luria-Bertani (LB) broth/agar was purchased from Himedia. HAuCl_4_, sodium citrate, and sodium chloride were supplied by the Merck Company.

***Methods***

*Bacterial culture*

Bacterial strains including EHEC, *S. typhi*, and *L. monocytogenes* were cultured at 37 ^°^C for 16 hr at the surface of plates containing LB agar. Then, the grown bacteria were used for the preparation of microbial suspension with turbidity equivalent to 3 McFarland, which contains approximately 10^9^ cells per ml. The suspension was serially diluted to 10^1^ CFU/ml and the accuracy of the prepared concentrations was tested by culturing the last three dilutions (10^3^, 10^2^, and 10^1^ CFU/ml). Concisely, 1 ml of each dilution was transferred to plate count agar and after aerobic incubation at 37 ^°^C for 16 hr, the number of colonies was counted manually. All bacterial concentrations were prepared in sterile distilled water (SDW).

*Preparation of AuNPs*

AuNPs with an average diameter of 13 nm were produced according to the literature (13, 22, 23). Briefly, in a 250 ml round bottom flask, 1 ml of HAuCl_4_ solution (50 mM) was added to 49 ml deionized DW. The mixture was heated under reflux conditions. As the contents of the flask began boiling, 1.94 ml sodium citrate (0.1 M) was added to the rapidly stirring solution. The system was kept to heat and reflux for another 20 min. Then, the heating was stopped but stirring was continued until the system reached room temperature (RT). The resulting AuNPs solution with burgundy red color was sampled to determine its quality using a particle size analyzer (Malvern, Nano ZS) and a transmission electron microscope (TEM) (Philips, CM120). Also, a sample was taken and diluted (1:10) for determining the absorbance at 520 nm to estimate the concentration of AuNPs through the Beer-Lambert equation.

*Investigation of the applicability of AuNPs in the presence of bacteria*

To understand whether the bacterial suspension could aggregate the AuNPs by itself, 50 µl of each microbial concentration was added to 100 µl of diluted nanoparticles in a 96-well plate.

Moreover, to examine whether the EHEC cells could protect AuNPs against salt aggregation via coating the nanoparticles, 20 µl of each concentration was inoculated in wells containing 130 µl of diluted AuNPs and incubated at RT. After 30 min, 50 µl of NaCl solution (400 mM) was supplemented and allowed to react for 5 min. The color change of each sample to blue was evaluated visually.

*Optimization of aptamer concentration to stabilize AuNPs solution*

Final concentrations of each aptamer including 0, 0.1, 0.25, 0.5, 1, and 2 µM were prepared in 190 μl of diluted AuNPs suspension. This suspension was incubated at RT for 30 min. Then, 50 μl of NaCl solution (500 mM) was added to each concentration and left to react completely. The lowest concentration having an absorption spectrum similar to the control (diluted AuNPs without NaCl) was selected as AuNPs stabilizing concentration.

*Optimization of NaCl concentration for AuNPs aggregation in the presence of aptamers*

Half of the stabilizing concentration of each aptamer obtained from the previous step was added to the wells containing diluted AuNPs suspension. After 30 min, the wells were treated with different quantities of NaCl solution (1 M). The lowest volume of NaCl solution which led to aggregation of AuNPs was recorded for each aptamer and the final concentration of NaCl in the well was calculated.

*Specificity evaluation of the aptamers*

The specificity test of the aptamers was performed against EHEC, *S. typhi, *and* L. monocytogenes*. The previous concentration of each aptamer was prepared in 100 µl AuNPs and incubated at RT. After 30 min, 10 µl of 10^6^ CFU/ml related to each bacterium prepared in SDW was separately added to the wells and incubated again for the same duration. Finally, the NaCl solution was added and incubated for 5 min. The absorption ratio of each bacterium (A700/A520) was compared with negative control (aptamer and AuNPs) and Δ(A700/A520) was plotted for all aptamers, individually. To have a better understanding of the applied aptamers, their secondary structure was predicted using the Mfold software (http://unafold.rna.albany.edu/).

*Determination of the linear range of biosensor, LOD, and LOQ*

According to the 0.5 McFarland turbidity, a bacterial suspension having approximately 10^8^ CFU/ml was prepared in SDW for EHEC. The accuracy of the concentrations was assessed by the plate count method. Then, the selected aptamer was added to various wells of a 96-well plate containing 100 µl AuNPs to reach the final concentration of 0.5 µM. Three controls were also considered: one with AuNPs and an aptamer, and two with AuNPs and SDW (instead of aptamer). Like the specificity test, the wells were incubated for 30 min and then 10 µl of each bacterial concentration was inoculated in separate wells. After incubation for another 30 min, 7 µl NaCl solution was added and set aside for 10 min. The first control containing aptamer as well as one of the other controls received the same quantity of NaCl solution while for the third control equal volume of SDW was added. The absorption ratio of each well (A700/A520) was compared with control (aptamer + AuNps) and Δ(A700/A520) for the logarithm of bacterial concentration was plotted. Based on the linear range of the plot, both LOD and LOQ were calculated.

## Results

*Bacterial culture*

Evaluation of the bacterial suspensions via plate count method revealed that the test tube containing ~10^2^ CFU/ml had approximately the same number of bacteria. Therefore, the estimated dilutions were considered correct.

*AuNPs characterization*

**Figure 1:**
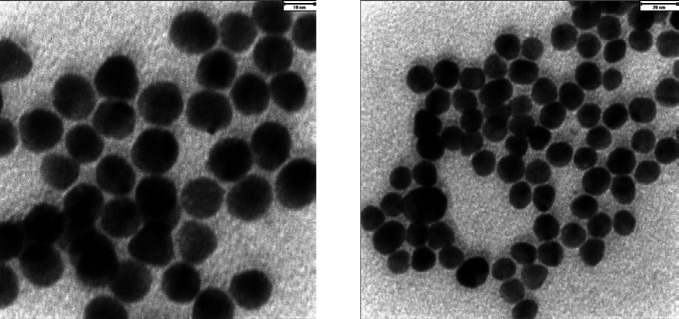
TEM images of produced AuNPs

The size distribution analysis of AuNPs indicated that the nanoparticles had an average diameter of ~13 nm (99.27%) with a poly-dispersity index (PdI) of 0.316 and zeta-potential of -34.8 mV. Moreover, TEM images confirmed the suitability of AuNPs size and distribution (Figure 1). Based on the Beer-Lambert equation, the concentration of the produced AuNPs was estimated as ~15 nM.

*Applicability of AuNPs in the presence of bacteria*

Unchanged color of diluted AuNPs against various microbial concentrations (10^9^ to 10 CFU/ml) indicated that these concentrations did not aggregate the nanoparticles. 

On the other hand, adding 10^9^ CFU/ml bacterial suspension to diluted AuNPs protects them from aggregation in the presence of NaCl solution. Therefore, the final concentration of each bacterium in the wells was adjusted below 10^8^ CFU/ml in the rest of the experiments.

*Optimization of aptamers concentration*

**Figure 2:**
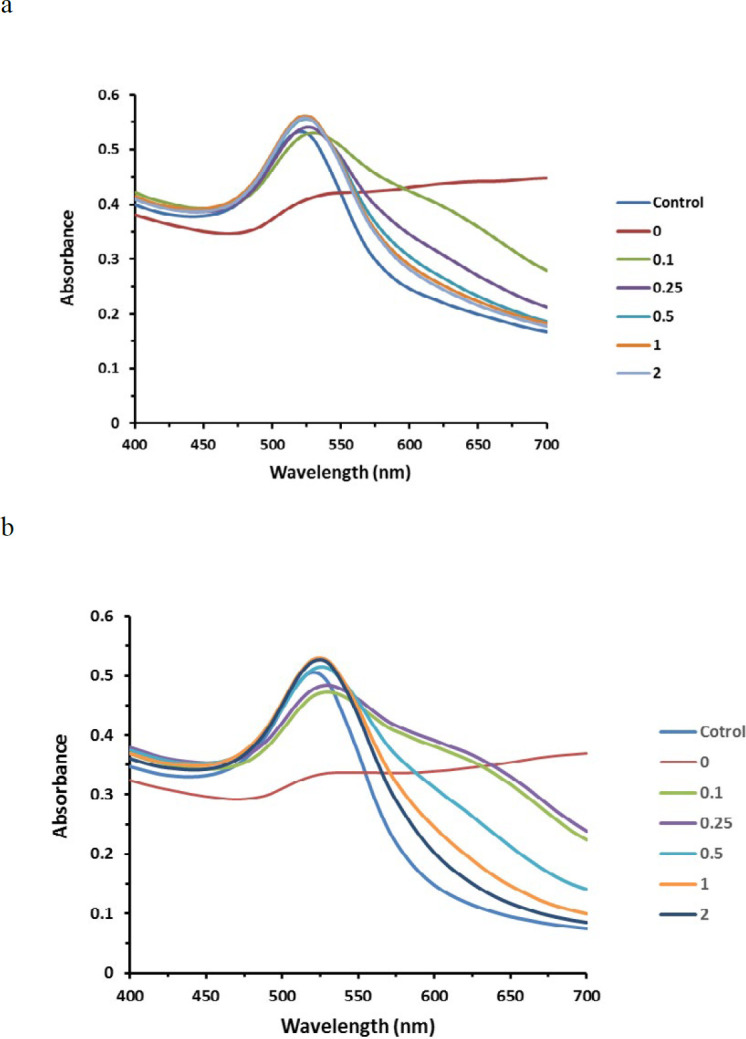
Changing of the absorption spectrum of AuNPs in the presence of various concentrations of aptamers after salt addition (final concentration of aptamer (µM) was inserted next to each graph, the control was considered as diluted AuNPs without NaCl) a) absorption spectrum for Apt 1, b) absorption spectrum for Apt 2 and 3. As clarified, the λ_max _of AuNPs shifted from 520 nm (control) to 700 nm (0) after salt addition. The first concentration of each aptamer that provides the absorption curve similar to the control was considered as the AuNPs stabilizing concentration

Between several dilutions of the aptamers in AuNPs suspension, the first concentration that protected nanoparticles against salt-induced aggregation and gave an absorption spectrum similar to the control was considered as the lowest stabilizing concentration (Figure 2). Thus, the final concentration of 0.5 µM was selected for aptamer 1 (Apt 1) and 1 µM was taken for aptamers 2 (Apt 2) and 3 (Apt 3) as the minimum amount of ssDNA required.

*Optimization of NaCl concentration*

To minimize aptamer consumption, half of their stabilizing concentration was added to AuNPs suspension and subsequently, various amounts of NaCl (1 M) were inoculated. Finally, for Apt 1 and 3, 6 µl (final concentration of ~50 mM) and for Apt 2, 8 µl (final concentration of ~70 mM) were recorded as the minimum volume of NaCl solution to aggregate AuNPs. Therefore, for the next steps, 1 µl less was utilized.

*Specificity evaluation of the aptamers*

**Figure 3:**
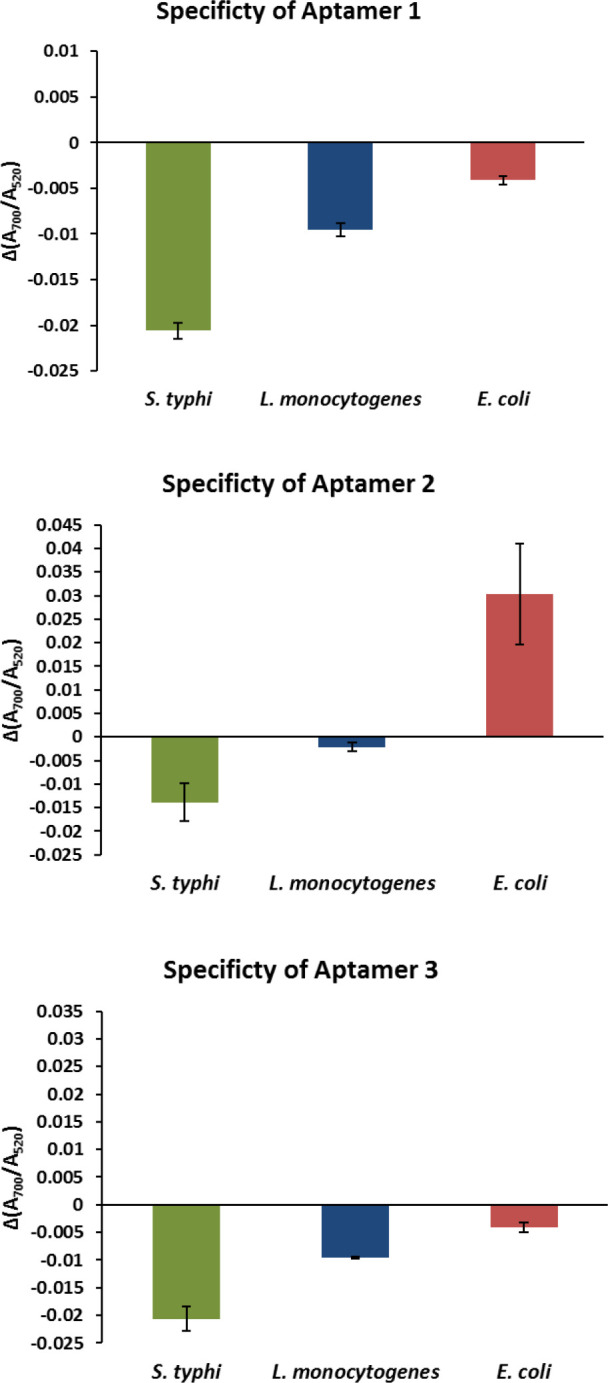
Specificity chart of aptamers for detection of EHEC against *Salmonella typhi* and *Listeria monocytogenes*

The specificity of the aptamers was evaluated in the presence of the optimized concentration of each one. All bacteria were inoculated individually to reach the dilution of ~10^5^ CFU/ml in the test wells. After addition of the optimized volume of the NaCl solution, comparison of the absorption ratio of the samples and the negative control (Δ(A700/A520)) revealed that only Apt 2 acted as an ideal aptamer for development of the biosensor (Figure 3).

*Determination of the linear range of biosensor, LOD, and LOQ*

**Figure 4.**
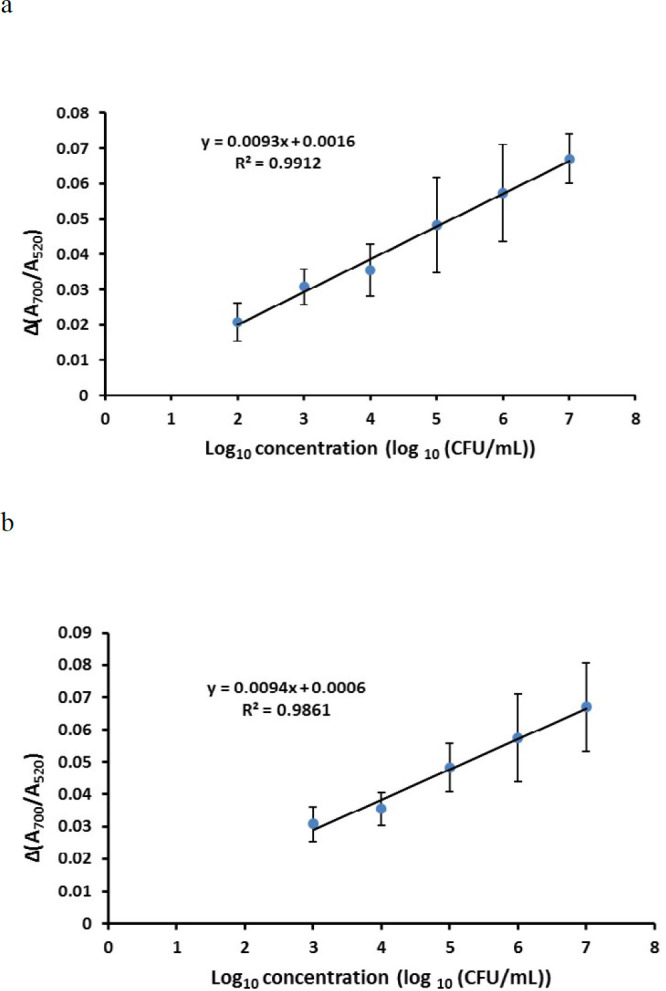
Calibration curve of EHEC aptasensor in water. Δ(A700/A520) indicates subtraction of absorption ratio (A700/A520) of each bacterial concentration form control (aptamer + AuNps). a) The primitive curve (before calculation of LOQ), b) The final calibration curve (after calculation of LOQ)

To determine the linear range of the aptasensor, serially diluted concentrations of EHEC were added separately to the wells containing Apt 2–modified AuNPs suspension. After incubation period and addition of the optimized volume of the NaCl solution, the aggregation intensity of AuNPs for each bacterial dilution was evaluated through the measurement of the difference between the absorption ratio of the samples and the first control (Δ(A700/A520)) (Figure 4a). The wells containing the other two controls showed blue and red colors, respectively. LOD was calculated from the following equation:

 LOD=3×SD (for control)/line slope

which was determined to be~263 CFU/ml. Therefore, the LOQ of the biosensor was estimated as 876 CFU/mL. The calibration plot was schemed again from the calculated LOQ (Figure 4b).

**Table 1:** The sequence of aptamers applied in this study for fabrication of aptasensor

**ssDNA**	**Sequence**	**Reference**
Apt 1	5ʹ-ATCCGTCACACCTGCTCTATCAAATGTGCAGATATCAAGACGATT TGTACAAGATGGTGTTGGCTCCCGTAT-3ʹ	(7, 13, 21)
Apt 2	5ʹ-ATCAAATGTGCAGATATCAAGACGATTTGTACAAGAT-3ʹ	(7)
Apt 3	5ʹ-CCGGACGCTTATGCCTTGCCATCTACAGAGCAGGTGTGACGG-3ʹ	(7, 13, 21)

**Figure 5:**
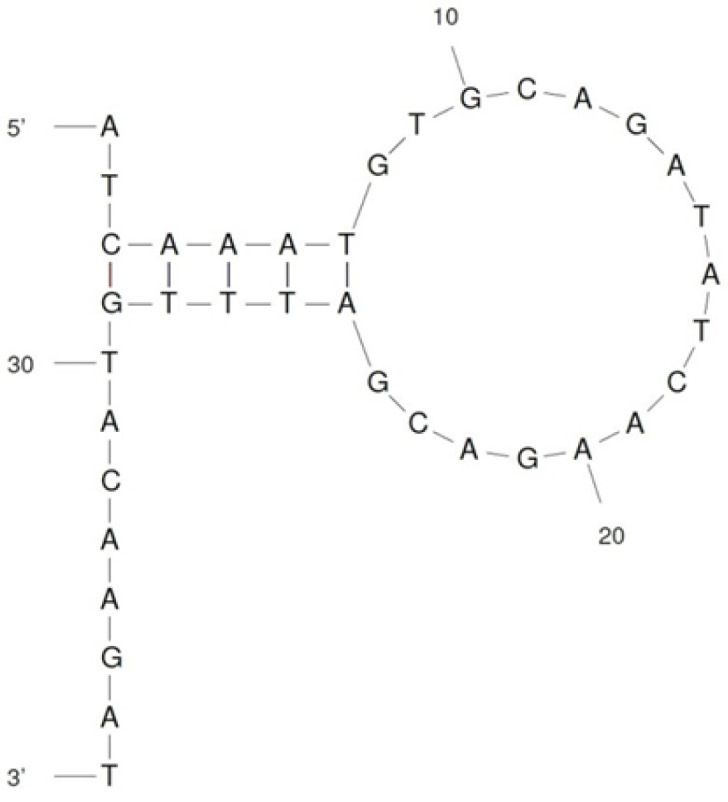
The predicted secondary structure of Apt 2 by Mfold software

**Table 2:** Comparison of current nanomaterial-based assay for determination of EHEC with some other biosensors

Method base	Recognition component	Transducer	LOD	Reference
Aptamer	Aptamer	Electrochemical	2 CFU/mL	(39)
Aptamer	Aptamer	Electrochemical	10^2^ CFU/mL	(9)
Aptamer	Aptamer	Piezoelectric effect	1.46 × 10^3^ CFU/mL	(40)
Aptamer	Aptamer	Chemiluminescence	4.5×10^3^ CFU/mL	(41)
Aptamer	Aptamer	Optical	10^4^ CFU/mL	(7)
Aptamer	Aptamer	Optical	10 CFU/mL	(13)
Aptamer	Aptamer	Fluorescence	10^2^ CFU/mL	(24)
Aptamer	Aptamer	Fluorescence	10^2^ CFU/mL	(42)
Immunoassay	Antibody	Fluorescence	5 × 10^2^ CFU/mL	(43)
Immunoassay	Antibody	Electrochemical	10 CFU/mL	(44)
Immunoassay	Antibody	Electrochemical	2.84 × 10^3^ CFU/mL	(45)
Immunoassay	Antibody	Optical	2.3 × 10 ^3^ CFU/mL	(46)
Immunoassay	Antibody	Optical	4.5 × 10^5^ CFU/mL	(47)
immunoassay	Antibody	Electrochemical	7.98 × 10^3^ CFU/mL	(48)
immunoassay	Antibody	Optical	1 CFU/mL	(49)
immunoassay	Antibody	Optical	1 × 10^4^ CFU/mL	(50)
Immunoassay	Antibody	Optical	100 CFU/mL	(51)
Immunoassay	Antibody	Optical	1.08×10^2^ CFU/mL	(52)
Immunoassay	Antibody	Electrochemical	10^2^ CFU/mL	(53)
Immunoassay	Antibody	Electrochemical	148 CFU/mL	(54)
Immunoassay	Antibody	fluorescence	<5 CFU/mL	(55)
Immunoassay	antibody	Fluorescence	10^5^ cells/mL	(56)
Immunoassay	Antibody	Chemiluminescence	10^5^ cells/mL	(57)
Immunoassay	Antibody	SPR signal	10^3^ cells/mL	(58)
Immunoassay	Antibody	Electrochemical	148 cells/mL	(54)
PCR	Oligonucleotide	Fluorescence	296 CFU/mL	(59)
Aptamer	Aptamer	Optical	236 CFU/mL	Current study

## Discussion

Infectious diseases due to viral, bacterial, or protozoan contamination are the main sources of human morbidity and mortality, surpassing cancer and cardiovascular diseases (15). Hence, detection, identification, and quantification of microbial organisms are vital subjects especially in the areas of clinical diagnosis, biodefense, food safety, and water and environmental analysis (24). Particularly, recreational waters are permanently monitored worldwide to protect people from enteric pathogens including *E. coli* and *Enterococcus* spp. (25).

Target detection in diagnostic tools and sensors depends on successful molecular recognitions. In 1990, the Gold Laboratory introduced an *in vitro* selection process, called Systematic Evolution of Ligands by EXponential enrichment (SELEX), which separates one or a few specific nucleic acid sequences (ssDNA or RNA) having high affinity and specificity to the user-defined considered targets and the resulting oligonucleotides are called aptamers (26, 27). Up to now numerous aptamers have been developed against various target molecules from metal ions and small molecules such as amino acids to macromolecules like proteins and polysaccharides, and even whole cells including pathogenic micro-organisms (18, 26).

In this study, we incorporated the specific recognition ability of an aptamer specific for EHEC to nanotechnology-based particles, AuNPs. The inimitable properties of nanomaterials that are completely different from a bulk counterpart in terms of size (1–100 nm), shape, and surface area result in higher surface reactivity, conductivity, and magnetic features (4, 28). Among several nanomaterials that have been fabricated, AuNPs have unique characteristics, such as unusual optical and electronic specifications, easy water dispersal, high stability, biological compatibility, and ability of surface functionalization (28, 29). They also have controllable morphology and size distribution besides easy modification of the surface via functional groups. Their surface plasmon resonance (SPR) feature which causes color changes from red (dispersed form) to blue (aggregate form) provides a powerful and easy-to-use colorimetric reporter. This alteration results from both scattering and dipole-dipole coupling between adjacent particles (28, 30). Therefore, the main advantage of AuNPs-based biosensors is the reflection of molecular events into color changes, which can be easily investigated by the naked eye (31). However, proteins can cover the surface of AuNPs and protect them from aggregation caused by salt (18). To investigate whether bacterial suspensions have similar protecting effects, various microbial dilutions were prepared and incubated with AuNPs. As the final concentration of 10^8^ CFU/ml displayed a protecting effect, further dilutions were used in other experiments.

Then the minimum concentration of aptamers stabilizing AuNPs against NaCl was detected. This concentration is influenced by various factors such as adenine and thymine content as well as aptamer length (29). As illustrated in Figure 2, the maximum absorption wavelength of unstabilized AuNPs shifted from 520 nm to 700 nm after salt addition, indicating nanoparticle aggregation. When the lowest stabilizing concentration of ssDNA was added, the absorption curve became similar to the control, which was determined as 0.5 µM for Apt 1 and 1µM for Apt 2 and 3. These concentrations were halved to reduce aptamer consumption. However, the minimum volume of NaCl solution (1 M) to aggregate AuNPs was determined.

Then, the specificity of each aptamer was evaluated. The inoculation of EHEC causes the specific aptamer to leave AuNPs surface and bind to EHEC cells. Subsequently, the naked AuNPs would aggregate upon addition of the NaCl solution and the red color of the wells containing EHEC turns to blue (the λ_max_ would shift from 520 nm to 700 nm). Especially for Apt 2, this color-changing was sharper in the presence of EHEC. This aptamer was used for biosensor fabrication based on unmodified AuNPs (Figure 5). 

Generally, during citrate reduction of HAuCl4, AuNPs are coated with citrate anions. In other words, the citrate anions act as both reducer and stabilizer during the AuNPs formation process. The excess citrate stabilizes the AuNPs via electrostatic repulsion which results from a complex multilayered assembly of adsorbed anions with various oxidation states (18, 29, 32). The addition of NaCl neutralizes the negative charges through Na^+^ cation and at a certain threshold concentration of Na^+^, nanoparticles become aggregated via hydrophobic and van der Waals forces. The presence of ssDNA adsorbed on the AuNPs surface, help to enhance the AuNPs’ negative charges and their stability. Consequently, more salt is needed to aggregate them (18, 23, 32). Under these conditions, the presence of target displaces the adsorbed aptamers competitively from AuNPs surface and makes them susceptible to salt-induced aggregation (18). This method has been used extensively in the literature for detection of various pathogens and microbial toxins including *Cronobacter sakazakii *(33), *Salmonella typhimurium *(34), human immunodeficiency virus (HIV) (35), staphylococcal enterotoxin B (36), aflatoxin B1 (37), and Ochratoxin A (38). The most important specification of this technique is the ability of AuNPs to perform assessments without labeling necessity. Absence of modifications can certify that the original conformation of aptamer is preserved when binding with its target, leading to high affinity and very sensitive and convenient detection (23). 

Finally, the linear range of the aptasensor response, LOD, and LOQ were determined. Since there was no difference in the recorded response between the final concentration of 10 and 10^2^ CFU/ml EHEC, the initial calibration curve was plotted between 10^2^ to 10^7^ CFU/mL. Based on this diagram, LOD (the lowest microbial concentration at which the aptasensor was able to detect but not quantify with N/S of 1:3) was calculated as~263 CFU/ml. Therefore, LOQ (the lowest bacterial concentration at which the aptasensor was able to detect and quantify with N/S of 1:10) was estimated as~876 CFU/ml (approximately 1000 CFU/ml). So, the calibration curve was plotted again from the logarithmic number of detectable EHEC.

Compared to conventional methods such as bacterial culture and incubation, the designed biosensor provides convenient detection in about 60 min. Moreover, LOD was comparable to many rapid detection methods without requirement of any sophisticated and expensive devices that complicate the detection process. Some similar research and their applied methods were summarized in Table 2.

## Conclusion

In the current study, a simple, rapid, direct and cost-effective detection was described for EHEC based on a short ssDNA aptamer (37 mer) and innate SPR property of AuNPs. The fabricated label-free aptasensor could quantify EHEC in a very small water sample (10 µl) well over the wide range of 876-10^7^ CFU/ml. However, LOD was good enough (236 CFU/ml) to identify EHEC contaminated water in about 1 hr. This method also avoids the aptamer labeling or AuNPs modification. Finally, the approach could be applied directly without complex instrumentation and pretreatment steps for running water, especially in areas with poor access to treated water and may also be valuable for both diagnostics and therapeutics.
